# A narrative review on trans-nasal pulmonary aerosol delivery

**DOI:** 10.1186/s13054-020-03206-9

**Published:** 2020-08-17

**Authors:** Jie Li, James B. Fink, Ronan MacLoughlin, Rajiv Dhand

**Affiliations:** 1 Division of Respiratory Care, Department of Cardiopulmonary Sciences, Rush University Medical Center, 1620 W Harrison St, Tower LL1202, Chicago, IL 60612 USA; 2 Aerogen Pharma Corp, San Mateo, CA USA; 3 Aerogen, Galway, Ireland; 4 Department of Medicine, University of Tennessee Graduate School of Medicine, Knoxville, TN USA

**Keywords:** High-flow nasal cannula, Aerosol therapy, Asthma, Chronic obstructive pulmonary disease, Pulmonary hypertension, Oxygen therapy, Jet nebulizer, Vibrating mesh nebulizer

## Abstract

The use of trans-nasal pulmonary aerosol delivery via high-flow nasal cannula (HFNC) has expanded in recent years. However, various factors influencing aerosol delivery in this setting have not been precisely defined, and no consensus has emerged regarding the optimal techniques for aerosol delivery with HFNC. Based on a comprehensive literature search, we reviewed studies that assessed trans-nasal pulmonary aerosol delivery with HFNC by in vitro experiments, and in vivo, by radiolabeled, pharmacokinetic and pharmacodynamic studies. In these investigations, the type of nebulizer employed and its placement, carrier gas, the relationship between gas flow and patient’s inspiratory flow, aerosol delivery strategies (intermittent unit dose vs continuous administration by infusion pump), and open vs closed mouth breathing influenced aerosol delivery. The objective of this review was to provide rational recommendations for optimizing aerosol delivery with HFNC in various clinical settings.

## Introduction

In severely hypoxemic patients, supplemental oxygen is routinely administered by high-flow nasal cannula (HFNC). HFNC is superior to conventional oxygen therapy in improving oxygenation and ultimately for avoiding intubation/reintubation in acutely ill patients [1–5]. Trans-nasal pulmonary aerosol delivery by HFNC combines the benefits of both HFNC and aerosol therapy [6, 7]. Since 2008, in vitro and in vivo studies have explored factors influencing delivery of aerosol with HFNC and the clinical effectiveness of this route of administration. In this article, we review the available evidence and provide a scientific basis for optimizing aerosol delivery with HFNC in various clinical settings.

## Literature search strategy and results

A search of the published English literature was conducted in PubMed, Medline, and Scopus until February of 2020, using the following keywords: (“high-flow nasal cannul*” OR “high flow cannul*” OR “high flow oxygen therapy” OR “high flow oxygen” OR “high flow therapy” OR “HFNC” OR “trans-nasal”) AND (“aerosol” OR “nebuliz*” OR “inhal*”). Publication types included in vitro/bench studies, scintigraphy studies for animal or healthy volunteers, clinical retrospective and prospective studies, randomized controlled trials, and questionnaire surveys. In total, databases identified 620 records and 42 original studies investigating aerosol delivery with HFNC were finally included. Articles were excluded for the following reasons: duplicates (153), did not investigate aerosol delivery via HFNC (415), conference abstracts (10), review articles (6), and letters (4).

## Clinical evidence of trans-nasal aerosol delivery

Trans-nasal aerosol delivery is increasingly employed in the intensive care units (ICUs). A survey of pediatric units in the USA reported that 75% of respondents employed trans-nasal aerosol delivery, while the remainder discontinued HFNC and used more conventional methods for delivering aerosols [8]. While demonstrating the popularity of aerosol delivery via HFNC in children, the survey also revealed concerns about its clinical efficacy. In Table 1, we summarize current clinical evidence regarding aerosol delivery with HFNC.

**Table 1:** Clinical studies using trans-nasal aerosol delivery via HFNC in adults and children

Author, year	Study type	Patient	Inhaled medication	Comparison	Finding
Bräunlich and Wirtz 2018 [9]	RCT crossover	Adults: 26 stable COPD	Salbutamol 2.5 mg + ipratropium 0.5 mg	JN via HFNC at 35 L/min vs JN alone	FEV_1_ change: 9.4 ± 13.6 vs 11.1 ± 17.2%, *p* = 0.5
Réminiac et al., 2018 [10]	RCT crossover	Adults: 25 stable patients with reversible airflow obstruction	2.5 mg albuterol	VMN via HFNC at 30 L/min vs JN with mask	FEV_1_ improvement: 0.33 (0.14, 0.39) vs 0.35 (0.18, 0.55) L, *p* = 0.11
Madney et al., 2019 [11]	RCT crossover	Adults: 12 stable COPD	5 mg salbutamol	VMN via HFNC at 5 L/min vs JN via HFNC	Urinary salbutamol excretion at 30 min and 24 h were higher with VMN than JN via HFNC (*p* < 0.05)
Li et al., 2019 [12]	Prospective dose response study	Adults: 42 stable asthma and COPD patients	Albuterol at an escalating dose of 0.5, 1.5, 3.5, and 7.5 mg	VMN via HFNC at 15–20 L/min vs MDI+Spacer	FEV_1_ increment at cumulative dose of 1.5 mg via HFNC was similar to 400 mcg albuterol via MDI+Spacer: 0.34 ± 0.18 vs. 0.34 ± 0.12 L, *p* = 0.878
Ammar et al., 2018 [13]	Retrospective	Adults: 29 patients with hypoxemia and PH	Epoprostenol	VMN via HFNC at 39 ± 11 L/min	PaO_2_/F_I_O_2_ improvement of 60 ± 50 mmHg
Li et al., 2019 [14]	Retrospective	Adults: 11 ICU refractory hypoxemia patients comorbid with PH and/or RVD	Epoprostenol	VMN via HFNC at 35–40 L/min	45.5% had SpO_2_/F_I_O_2_ improvement > 20%
Li et al., 2020 [15]	Retrospective Cohort comparison	Adults: 51 ICU patients with PH and/or RVD	Epoprostenol	VMN via HFNC at constant flow (*n* = 26) vs flow titrated based on individual response to inhaled epoprostenol (*n* = 25)	The percentage of patients who met the criteria for a positive response was higher in the flow titration group compared to the group with constant flow (85.7% vs. 50%, *p* = 0.035).
Morgan et al., 2015 [16]	Retrospective	Pediatrics: 5 infants acute bronchiolitis with respiratory distress	Albuterol	VMN via HFNC at 5–8 L/min vs JN and face mask	Compared to JN with mask, HR increment was higher after inhaling albuterol with VMN via HFNC; patient agitation was improved
Valencia-Ramos et al., 2018 [17]	RCT crossover	Pediatrics: 6 infants with bronchiolitis	Albuterol	VMN via HFNC around 8 L/min vs JN with mask	Increased level of comfort and satisfaction
Al-Subu et al., 2020 [18]	Retrospective	Pediatrics: 28 children with asthma or bronchiolitis	Albuterol	VMN via HFNC at 2–4 L/min vs VMN with mask	HR increased by 9.98 (95% CI 3.72–16.2) with VMN via HFNC vs 0.64 (95% CI, 1.65–2.93) beats/min with VMN via mask (p < 0.001)
Baudin et al., 2017 [19]	Retrospective	Pediatrics: 39 status asthmaticus (10 had severe acidosis at admission)	Albuterol	VMN via HFNC at maximum 1 L/kg/min vs standard oxygen without HFNC	In HFNC group, HR (165 ± 21 vs. 141 ± 25/min, *p* < 0.01) and RR (40 ± 13 vs. 31 ± 8/min, *p* < 0.01) decreased, and blood gas improved in the first 24 h

*HFNC* high-flow nasal cannula, *JN* jet nebulizer, *FEV*_*1*_ forced expiratory volume at the first second, *COPD* chronic obstructive pulmonary disease, *MDI* metered dose inhaler, *RCT* randomized controlled trial, *VMN* vibrating mesh nebulizer, *PH* pulmonary hypertension, *RVD* right ventricular dysfunction, *HR* heart rate, *RR* respiratory rate, *PaO*_*2*_ partial pressure of arterial oxygen, *SpO*_*2*_ peripheral capillary oxygen saturation, *F*_*I*_*O*_*2*_ fraction of inspired oxygen, *CI* confidence interval

### Adult patients: inhaled albuterol delivery via HFNC

In 2018, Bräunlich and colleagues reported that 26 patients with stable chronic obstructive pulmonary disease (COPD), who inhaled 2.5 mg albuterol and 0.5 mg ipratropium via a small volume jet nebulizer (JN) and mouthpiece or in-line with HFNC (TNI medical AG, Wuerzburg, Germany) at a gas flow of 35 L/min, had similar bronchodilator effect (*p* = 0.5) [9]. Likewise, Réminiac and colleagues compared delivery of 2.5 mg albuterol with a vibrating mesh nebulizer (VMN) (Aerogen Solo, Aerogen, Ireland) via HFNC (Airvo2, Fisher & Paykel, New Zealand) versus a JN with mask in a cross-over RCT in 25 stable patients with reversible airflow obstruction and reported similar improvements in forced expiratory volume in the first second (FEV_1_) (*p* = 0.11) [10]. In a crossover RCT with 12 stable COPD patients, Madney and colleagues compared systemic bioavailability of albuterol administered by JN or VMN in line with HFNC at 5 L/min. Urinary albuterol excretion at 30 min and 24 h was 2-fold higher with the VMN than the JN (*p* < 0.05) [11].

**Fig. 1:**
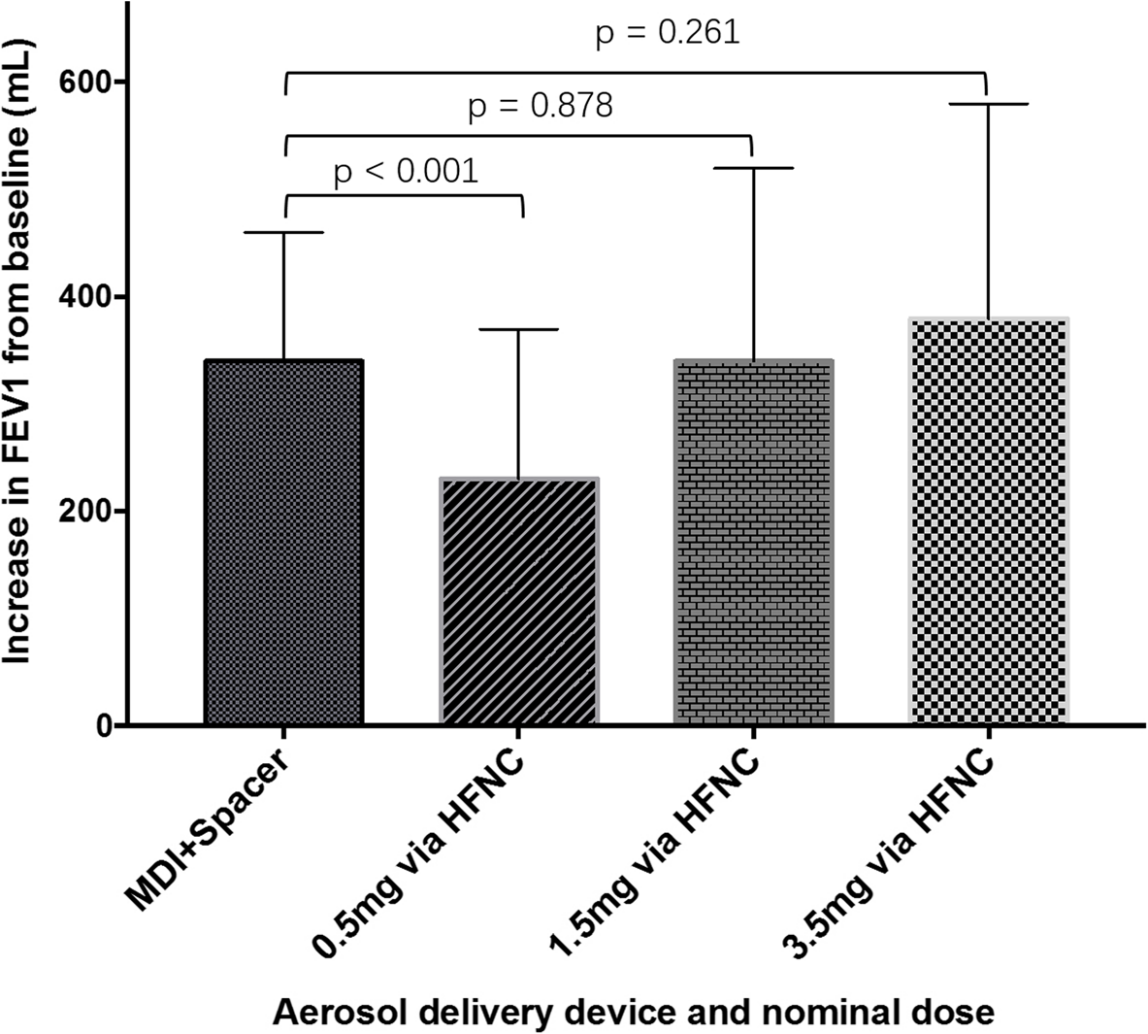
Improvement in FEV_1_ (mL) from baseline after inhalation of 400 mcg albuterol via MDI with spacer or doubling doses of albuterol via VMN with HFNC (cumulative doses of 0.5, 1.5, and 3.5 mg). Figure modified from reference [12]. In 42 bronchodilator responsive patients with asthma or COPD, FEV_1_ improvement after administration of 400 mcg albuterol via MDI and spacer was higher than that after inhalation of 0.5 mg albuterol via VMN with HFNC, but similar to that observed after inhalation of cumulative doses of 1.5 mg or 3.5 mg of albuterol via VMN with HFNC. COPD, chronic obstructive pulmonary disease; MDI, metered dose inhaler; FEV_1_, forced expiratory volume in the first second; VMN, vibrating mesh nebulizer; HFNC, high-flow nasal cannula

The label dose of albuterol solution in the USA and Europe is 2.5 mg. Li and co-workers performed a dose-response relationship study in 42 stable asthma and COPD patients with known positive responses to 400 mcg albuterol via metered dose inhaler (MDI) and spacer. The subjects inhaled escalating doubling doses via VMN and HFNC with gas flow of 15–20 L/min. The improvement of FEV_1_ at the cumulative dose of 1.5 mg with VMN and HFNC was similar to that with MDI and spacer (*p* = 0.878) (Fig. 1) [12].

### Adult patients: inhaled epoprostenol delivery via HFNC

Inhaled epoprostenol, a pulmonary vasodilator, has been used off-label for several decades to treat mechanically ventilated patients with pulmonary hypertension and/or refractory hypoxemia [20, 21]. In two small retrospective studies, adult patients with pulmonary hypertension and refractory hypoxemia improved oxygenation after inhaling epoprostenol via HFNC at an average gas flow of 40 L/min [13, 14]. Mean pulmonary arterial pressure was reduced more effectively by titrating HFNC gas flow based on individual response to inhaled epoprostenol at the bedside compared with a constant HFNC flow [15]. Future prospective studies with larger sample size are needed to validate these findings.

### Pediatric patients: inhaled albuterol delivery via HFNC

In 2015, Morgan and colleagues studied five infants with acute bronchiolitis and respiratory distress unresponsive to three treatments with JN via mask [16]. After inhaling albuterol via VMN and HFNC, infants appeared markedly more comfortable, suggesting that albuterol administration with HFNC was beneficial. An observed increase in heart rate probably reflected delivery of a higher albuterol dose via VMN and HFNC. Likewise, in children receiving albuterol by VMN via HFNC with flow at 2–4 L/min or via face mask, the heart rates increased by 10 beats/min after inhaling albuterol via HFNC (*p* < 0.001 vs mask) [18]. In a cross-over RCT in 6 infants with bronchiolitis, albuterol delivery via VMN with HFNC (~ 8 L/min) improved patients’ comfort and satisfaction with treatment compared to JN and mask [17].

In a retrospective study of 39 children with status asthmaticus (10 had severe acidosis with pH < 7.30) who failed ≥ 3 treatments with nebulized albuterol via JN, intermittent boluses of albuterol delivered via HFNC at a maximum flow of 1.0 [0.8–1.1] L/kg/min were considered as a contributor in avoiding intubation [19].

### Summary

The standard bronchodilator doses delivered via HFNC at 15–35 L/min for adults and 1 L/kg/min for children generated similar clinical responses to those delivered with conventional aerosol devices. Further studies need to quantify aerosol delivery efficiency in critically ill patients.

## Factors influencing trans-nasal aerosol delivery

Since 2008, 18 in vitro [22–39] and 4 in vivo scintigraphy studies [38, 40–42] investigated factors influencing aerosol delivery via HFNC.

### Aerosol generator: VMN vs JN

When a JN is placed in-line with HFNC, the total gas flow in the HFNC system is greater than 6 L/min, which is the minimal flow to operate the JN. This flow requirement limits the use of JN via HFNC for infants and small children, who require HFNC flow ≤ 6 L/min. Moreover, JN integrated into a HFNC system may be contraindicated in systems that incorporate their own flow generators (e.g., Airvo 2 from Fisher & Paykel) as it alters oxygen, total flow, and pressure. In contrast, VMNs are driven by electricity with no additional gas flow required. Additionally, the residual volume of drug remaining in nebulizers is higher in JN than VMN (45% vs 3%) [41, 43]. Consequently, VMN generated 2–3 times higher inhaled dose than JN via HFNC for both pediatric and adult populations (Table 2) [11, 33, 38, 41]. For these reasons, VMNs are preferred over JNs for aerosol delivery with HFNC [8].

**Table 2:** Comparisons of inhaled dose between VMN and jet nebulizer via HFNC

Publication	Study type	Population	Flow (L/min)	Inhaled dose (%)	
				JN	VMN
Réminiac et al., 2017 [38]	In vivo	Infant	8	0.03 ± 0.03	0.09 ± 0.04
	In vitro			0.46 ± 0.12	0.52 ± 0.23
Ari, 2019 [33]	In vitro	Infant	6	1.45 ± 0.10	2.35 ± 0.30
		Pediatric	6	2.46 ± 0.10	5.37 ± 0.70
Madney et al., 2019 [11]	In vivo	Adult	5	7.90 ± 3.10	12.20 ± 4.40
Dugernier et al., 2017 [41]	In vivo	Adult	30	1.0 (0.70–2.0)	3.60 (2.10–4.40)

*VMN* vibrating mesh nebulizer, *JN* jet nebulizer, *HFNC* high-flow nasal cannula

### Aerosol carrier

HFNC gas functions as the “carrier” for aerosol, so that gas flow rate, gas density, and humidity could affect aerosol delivery efficiency.

### HFNC gas flow and patient’s inspiratory flow

In patients receiving HFNC therapy, the total inhalation flow is a combination of the patient’s inspiratory flow and HFNC gas flow. The contribution of each flow influences the efficiency of aerosol delivery. When VMN was utilized to deliver aerosol via HFNC during *quiet breathing*, aerosol deposition was inversely related to the gas flow (Table 3) [23, 26, 27, 33, 38, 40, 42, 44]. Turbulence generated with higher gas flow leads to greater impaction losses of the aerosol particles ≥ 3 μm during their passage through the cannula, prongs, and upper airways, thereby reducing the dose of aerosol delivered to the patient’s lower airway. Consequently, one guideline recommends reducing HFNC gas flow to 4 L/min during aerosol delivery to children [45].

**Table 3:** Studies comparing different gas flow settings for trans-nasal aerosol delivery with HFNC

Patient	Study type	Author	Nebulizer position	Collection filter placement	Breathing pattern	Inspiratory flow (IF)	Gas flow (GF)	GF: IF	Inhaled dose (%)
Adult	In vitro	Réminiac et al., 2016 [26]	Inlet of humidifier	Trachea	Quiet breathing: Vt 500 mL, RR 15 bpm, I:E = 1:1, Ti 2 s	15	30.0	2.0	6.70
							45.0	3.0	3.50
							60.0	4.0	3.0
					Distressed breathing: Vt 750 mL, RR 30 bpm, I:E = 1:1, Ti 1 s	45	30.0	0.67	10.30
							45.0	1.0	6.70
							60.0	1.33	5.10
		Dailey et al., 2017 [27]	Inlet of humidifier	Nasal prongs	Quiet breathing: Vt 500 mL, RR 16 bpm, I:E = 1:2, Ti 1.25 s	24	10.0	0.42	26.70 ± 1.30
							30.0	1.25	11.60 ± 1.20
							50.0	2.08	3.50 ± 0.20
					Distressed breathing: Vt 750 mL, RR 30 bpm, I:E = 1:1, Ti 1 s	45	10.0	0.22	13.0 ± 3.0
							30.0	0.67	33.0 ± 5.0
							50.0	1.11	25.0 ± 2.0
		McGrath et al., 2019 [44]	Outlet of humidifier	Trachea	Quiet breathing: Vt 500 mL, RR 15 bpm, I:E = 1:1, Ti 2 s	15	10.0	0.67	5.35 ± 2.81
							40.0	2.67	2.56 ± 1.38
							60.0	4.0	1.01 ± 0.26
	In vivo	Alcoforado et al., 2019 [42]	Inlet of humidifier	NA	Normal healthy volunteer, quiet breathing (*n* = 23)	NA	10.0	NA	17.23 ± 6.78
							30.0	NA	5.71 ± 2.04
							50.0	NA	3.46 ± 1.24
Pediatric	In vitro	Ari et al., 2011 [23]	Inlet of humidifier	Nasal prong	Infant quiet breathing: Vt 100 ml, RR 20 bpm, I:E 1:2	6	3.0	0.5	10.65 ± 0.51
							6.0	1.0	1.95 ± 0.50
		Réminiac et al., 2017 [38]	Inlet of humidifier	Trachea	Infant quiet breathing: Vt 25 mL, RR 40 bpm, I:E 1:2	3	2.0	0.67	4.15 ± 1.75
							4.0	1.33	3.29 ± 1.70
							8.0	2.67	0.52 ± 0.23
		Ari, 2019 [33]	Inlet of humidifier	Trachea	Child quiet breathing: Vt 250 mL, RR 20 bpm, Ti 1 s	15	4.0	0.27	8.64 ± 1.20
							6.0	0.40	5.37 ± 0.70
					Infant quiet breathing: Vt 100 mL, RR 30 bpm, Ti 0.7 s	8.6	4.0	0.47	3.27 ± 0.40
							6.0	0.70	2.35 ± 0.30
	In vivo	Réminiac et al., 2017 [38]	Inlet of humidifier	NA	Macaque (*n* = 3)	NA	2.0	NA	0.85 ± 0.57
							4.0	NA	0.49 ± 0.44
							8.0	NA	0.09 ± 0.04
		Corcoran et al., 2019 [40]	After a corrugated tubing segment	NA	Infants (*n* = 18)	NA	2.0	NA	4.50 ± 2.20
							0.2	NA	33.50 ± 13.0

*HFNC* high-flow nasal cannula, *Vt* tidal volume, *Ti* inspiratory time, *RR* respiratory rates, *I:E* ratio of inspiratory to expiratory time, *NA* not available

**Fig. 2:**
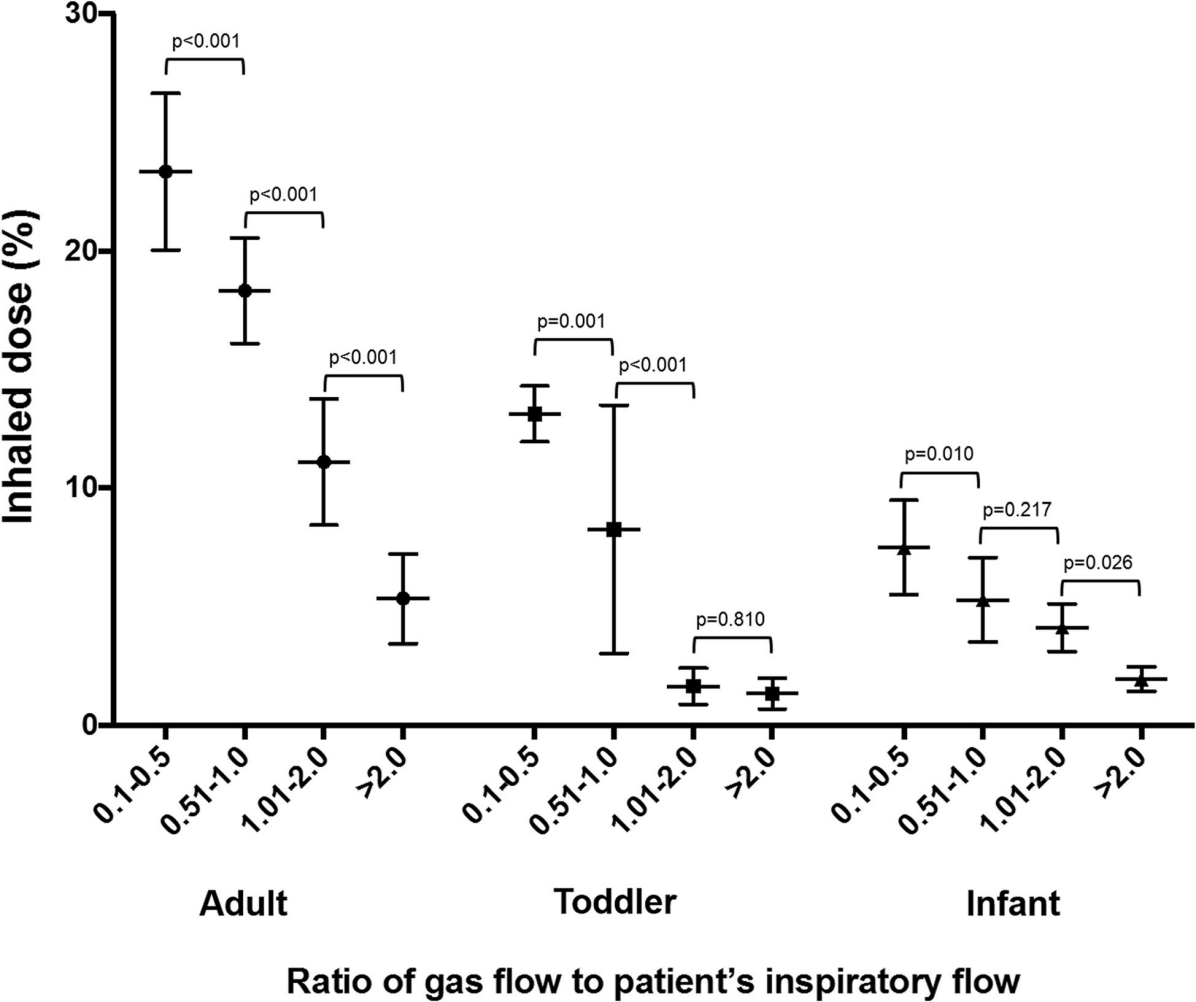
The relationship between inhaled dose and the ratio of HFNC gas flow to patient’s inspiratory flow in adult, toddler, and infant models. Mean and (±) SD values are shown. Figure modified from references [29, 30]. In adult, toddler, and infant in vitro models, as the ratio of HFNC gas flow to patient’s inspiratory flow increased, the delivered dose decreased, with a steep decline in aerosol delivery when HFNC gas flow was more than 2-fold higher than the patient’s inspiratory flow. Inhaled dose peaked when the HFNC gas flow was 0.1–0.5 of the patient’s inspiratory flow. For illustration, data from ratios of 0.1–0. 5, 0.51–1.0, 1.01–2.0, and > 2.0 in the original studies have been combined for this graphic. HFNC, high-flow nasal cannula

In contrast, during simulated *adult distressed breathing*, two in vitro studies reported that inhaled aerosol dose increased when gas flow decreased from 50 to 30 L/min [26, 27] and decreased when gas flow was reduced to 10 L/min [27]. Inhaled doses were higher during distressed breathing than quiet breathing with gas flows of 30 and 50 L/min [26, 27], but not at 10 L/min [27]. Subsequently, Li and colleagues utilized 5 different gas flows (5–60 L/min) and 6 different adult breathing patterns in their in vitro study and reported that the ratio of HFNC flow to patient’s inspiratory flow was more important than HFNC flow alone [29]. Inhaled drug dose was higher when gas flow was set below the patient’s inspiratory flow compared to gas flow exceeding inspiratory flow, and plateaued when HFNC flow was set at ~ 50% of the inspiratory flow [29]. These findings were consistent with a report in infants and children (Fig. 2) [30] and formed the basis for a RCT to compare albuterol delivery and effective dose at 3 different gas flow settings with a HFNC in patients with COPD or asthma [46].

Currently, no commercial device provides breath-by-breath measurement of patient’s inspiratory flow during HFNC. However, findings on the gas flow to patient’s inspiratory flow ratio [29, 30] should remind clinicians to titrate gas flow settings when employing HFNC for aerosol delivery, especially for drugs such as inhaled epoprostenol that produce immediate clinical responses. In support of this recommendation, a retrospective study in patients with pulmonary hypertension and hypoxemia found that titration of gas flow at the bedside led to a better response to inhaled epoprostenol via HFNC compared with application of a constant gas flow [15].

#### Gas density: oxygen vs heliox

Heliox (mixture of helium and oxygen) has lower density than oxygen or air and passes through narrow circuits and airways with less turbulent flow than oxygen. Heliox is employed to reduce airway pressures and gas trapping during severe airway obstruction. A meta-analysis reported that heliox provides potential short-term benefits for children with moderate to severe croup [47]. Reducing turbulence with heliox enhances aerosol delivery with HFNC, as previously reported in bench models of mechanical ventilation [48].

In pediatric [23] and adult [27] manikins, aerosol delivery efficiency using heliox showed limited superiority over oxygen only when HFNC gas flow exceeded patient’s inspiratory flow [27]. Using heliox as the carrier gas for the sole purpose of increasing aerosol therapy delivery is not cost-effective unless heliox is used to relieve dyspnea in patients with severe airway obstruction [47].

#### Dry vs heated humidified gas

In vitro and in vivo studies on mechanically ventilated patients noted that humidification reduced aerosol delivery to the lung [49, 50]. Interestingly, during trans-nasal aerosol delivery with flows ≥ 30 L/min, Alcoforado and co-workers found 1–1.5 times higher inhaled dose with dry than humidified gas [42]. Clinically, patient discomfort and potential adverse effects with nasal administration of dry gas at flows greater than 6–10 L/min should be considered. Moreover, turning off the humidifier for 30 min prior to aerosol administration during mechanical ventilation did not improve delivery efficiency [51]. For these reasons, the administration of dry gas in non-humidified circuits to deliver aerosol for prolonged periods should be discouraged in clinical practice.

### Nebulizer placement: close to patient vs at the inlet of humidifier

Both pediatric [25, 30] and adult [26, 39] in vitro studies reported that aerosol deposition with VMN placed at the inlet of humidifier was greater than with the nebulizer placed close to patient. The exception was in infants with extremely low gas flow (≤ 0.25 L/kg/min) where nebulizer placement closer to the patient was more efficient [30]. With the VMN placed farther away from the patient, “carrier” gas flow (including delivery gas flow and patient’s inspiratory flow, combined with low tidal volume) was probably insufficient to transport aerosol to the patient before aerosol sedimentation occurred.

### Open mouth vs closed mouth breathing

Open mouth breathing reduced inhaled dose compared to closed mouth breathing in the adult manikin during aerosol delivery with HFNC when gas flow was set higher than the patient’s inspiratory flow [26]. This observation was consistent with a report by Li and co-workers in a pediatric model [37]. Interestingly, when gas flow was lower than the patient’s inspiratory flow, open mouth breathing resulted in a higher inhaled dose than closed mouth breathing [37]. Perhaps aerosol carried with low gas flow collected in the nasal cavity during exhalation via mouth was drawn in with the next inhalation. In contrast, higher gas flows flushed the aerosol from the nasopharynx, thereby reducing the amount of drug available for the next inhalation [37].

### Delivery technique

#### Continuous administration using infusion pump vs unit dose

Clinically, aerosol therapy in the acute care setting involves either (1) intermittent unit dose delivery or (2) continuous aerosol delivery. For treatment of severe airway obstruction, administration of larger doses as frequently as every 15 mins over several hours is resource and labor intensive. Initially, “continuous” aerosol delivery was employed to administer high-dose short-acting bronchodilators for prolonged periods, conventionally using a large volume JN with facemask. However, noisy JN operation and cool aerosols produced by them can irritate young patients, causing them to cry during aerosol treatment, which significantly reduces the inhaled dose [52]. In contrast, in-line placement of VMN with active humidification and HFNC provides warm and humidified gas; aerosol generation is silent and significantly improves patients’ comfort and tolerance [16, 17].

Continuous administration of aerosol using VMN involves a pump feed to control rate and volume of dose emitted. At lower pump feed rates, duration between drops of medication reaching the mesh and producing aerosol is longer. Consequently, “continuous” administration has intermittent bursts of aerosol followed by periods of no aerosol. Li and colleagues reported that inhaled dose with unit dose delivery nebulizing continuously was higher than a similar nominal dose administered via infusion pump at low feed rate during the first 15 minutes of trans-nasal aerosol delivery, independent of gas flow settings [37]. This observation could be due to asynchrony of patient’s inhalation with intermittent aerosol production when individual drops reach the mesh during low-rate infusion pump delivery.

#### High vs low albuterol concentration

In the same study, inhaled dose with albuterol in high concentration was greater than with low concentration whether given by unit dose or infusion pump, with exception of lower delivery with high gas flow (2 L/kg/min) during infusion pump delivery [37].

### Aerosol generation: breathing synchronized vs continuous

Continuous generation of aerosol by nebulizers JN or VMN, in-line with HFNC, results in wastage to the atmosphere during the expiratory phase. Synchronized aerosol generation with patient’s spontaneous breathing increases inhaled dose during both invasive [53] and noninvasive ventilation [54, 55]. With a prototype breath-synchronized VMN, Li and colleagues reported inhaled dose was higher with breath-synchronized versus continuous aerosol generation when placed close to the patient with HFNC gas flow ≥ 10 L/min. However, when placed at the inlet of the humidifier, breath-synchronized VMN generated a higher inhaled dose than continuous operation only when HFNC gas flow was below 50% of patient’s inspiratory flow [39]. This finding is likely explained by storage of the aerosol in the HFNC circuit during the exhalation phase. The optimal ratio of HFNC gas flow to patient’s inspiratory flow that generates the highest inhaled dose depends on the balance between aerosol storage and gravitational sedimentation in the circuit.

### Other aerosol delivery methods during HFNC treatment

Alternatives to placing nebulizers in-line to administer aerosol during HFNC include placing the nebulizer with mouthpiece/mask over the nasal cannula; or discontinuing HFNC to administer aerosol by conventional methods [8].

#### Nebulizer or MDI+spacer with vs without concurrent HFNC

Administration of conventional aerosol devices (JN, VMN, or MDI with spacer) using mask/mouthpiece during HFNC reduced inhaled dose to a level that was only 6–50% of the inhaled dose with those devices alone without concurrent HFNC [32, 34], as high velocity gas from HFNC disperses aerosol away from the upper airway.

#### Aerosol delivery via HFNC vs conventional aerosol delivery

Aerosol delivery via HFNC at high gas flows (50 L/min for adult and 2 L/kg/min for children) generated similar inhaled dose as a JN and mask [33, 37, 38], but lower inhaled dose than VMN with mask [32, 33]. However, at lower gas flows (0.25–0.5 L/kg/min for pediatrics), the inhaled dose via HFNC was higher than that with VMN and mask [37] and 2–3-fold higher than that with JN and mask (Table 4) [37, 38].

**Table 4:** In vitro studies compared aerosol delivery via HFNC vs conventional aerosol device (JN or VMN with mask)

Author, year	Patient	HFNC gas flow setting (L/min)	Flow setting for conventional nebulizer (L/min)	Inhaled dose (%)		
				Aerosol delivery via HFNC	JN with mask	VMN with mask
Ari, 2019 [33]	Child	6	6	5.37 ± 0.7	5.76 ± 0.10	11.26 ± 1.90
		4		8.64 ± 1.2		
	Infant	6	6	2.35 ± 0.3	3.83 ± 0.50	7.20 ± 0.60
		4		3.27 ± 0.4		
Li et al., 2019 [37]	Child	25	8	2.84 ± 0.20	2.99 ± 0.41	3.65 ± 0.16
		3.75	2	11.57 ± 0.43	NA	3.82 ± 0.07
Réminiac et al., 2017 [38]	Infant	8	6	0.09 ± 0.04	0.71 ± 0.23	NA
		4		0.49 ± 0.44		
		2		0.85 ± 0.57		
	Toddler	8	6	0.52 ± 0.33	1.66 ± 0.06	NA
		4		3.29 ± 1.70		
		2		4.15 ± 1.75		
Bennett et al., 2019 [32]	Adult	50	8	6.81 ± 0.45	9.07 ± 0.26	NA
			6	NA	NA	36.21 ± 0.78

*HFNC* high-flow nasal cannula, *JN* jet nebulizer, *VMN* vibrating mesh nebulizer; NA, not available

### Other considerations in the in vitro studies

#### Airway model and placement of collecting filter

During in vitro studies, aerosol deposition was lower with collecting filter placed at “trachea” level [26, 32] than with filter placed distal to the nasal cannula (Table 5) [27]. This is because the anatomical volumes and structures of the upper airway serve as baffles upon which aerosol impacts, and the “exhalable” fraction of aerosol that stays in the trachea and upper airway at the end of inspiration is exhaled with the filter placed at “trachea.” Thus, results from in vitro studies especially with collecting filter placed close to nasal cannula could overestimate the actual aerosol drug delivery in vivo [38].

**Table 5:** Comparisons of the results with collecting filter placed at trachea vs nasal cannula in adult in vitro studies

Studies	Population	Breathing pattern	HFNC flow (L/min)	Inhaled dose (%)	
				Trachea	Nasal cannula
Réminiac et al., 2016 [26], and Dailey et al., 2017 [27]	Adult	Distressed breathing Vt 750 mL, RR 30 bpm, I:E = 1:1, Ti 1 s, inspiratory flow 45 L/min	30	10.3	13.0 ± 3.0
			45	6.7	33.0 ± 5.0
			60	5.1	25.0 ± 2.0
Bennett et al., 2019 [32], and Dailey, 2017 [27]	Adult	Quiet breathing: Vt 500 mL, RR 15 bpm, I:E = 1:1, Ti 2 s, inspiratory flow 15 L/min	10	5.4 ± 2.8	26.7 ± 1.3

*HFNC* high flow nasal cannula, *Vt* tidal volume, *RR* respiratory rates, *Ti* inspiratory time, *I:E* ratio of inspiratory time to expiratory time

#### Breathing profiles

No studies have fully characterized patients’ breathing profiles during HFNC treatment. Breathing parameters in the in vitro studies do not truly reflect patients’ breathing patterns, which vary breath by breath in individual and also display inter- and intra-patient variability [56].

#### Safety of trans-nasal aerosol on the nasal epithelium

The potential toxicity or harms of aerosol deposition in the nasopharynx during HFNC are unknown. For example, hypertonic saline, tobramycin solution, and dry air decrease ciliary beat frequency [57]. Elucidation of in vivo nasal toxicity with each drug formulation used with HFNC is necessary because many drugs are approved for delivery by nebulizer via facemask, with consequent potential for nasal exposure.

#### Environmental contamination

During aerosol delivery via HFNC, aerosol leakage from the nasal cannula to the environment combines with aerosol exhaled by patients into the atmosphere. Environmental fugitive emissions decreased as HFNC gas flow increased [44], likely due to turbulence effects of the high velocity gas leading to high impactive losses of aerosol *en route*. Bedside clinicians should employ personal protection during trans-nasal aerosol delivery, particularly when high-risk medications are administered.

### Summary

Among these in vitro studies, the ratio of HFNC gas flow to patient’s inspiratory flow was critical; optimal inhaled dose was achieved when HFNC gas flow was set ~ 50% of inspiratory flow. VMN used with HFNC generated a higher inhaled dose than JN. VMN placed at the inlet of humidifier generated a greater inhaled dose than VMN placed closer to the patient. When using dry gas or heliox as HFNC carrier gas, compared with humidified gas or oxygen, respectively, the inhaled dose was higher only when HFNC gas flow exceeded patient’s inspiratory flow. However, patient’s inability to tolerate dry gas or the high cost of using heliox particularly for prolonged duration is a deterrent to their routine use. Removing HFNC to use a conventional aerosol device did not improve drug delivery, and placing a conventional aerosol device via mask/mouthpiece concurrent with HFNC reduced drug delivery.

## Clinical implications and recommendations: trans-nasal aerosol delivery strategies for different patients

Table 6 provides recommendations on trans-nasal pulmonary aerosol delivery with HFNC, to help optimize aerosol delivery concurrent with HFNC.

**Table 6:** Recommendations on the use of trans-nasal aerosol pulmonary delivery

Techniques for aerosol delivery with HFNC	Recommendations	Evidence resource
Aerosol generator	VMN is more efficient than jet nebulizer when placed in-line with HFNC	In vitro pediatric [33, 38] In vivo adult [11, 41]
Discontinue HFNC treatment to deliver conventional aerosol treatment	Not recommended.	Adult in vivo [9, 10, 12] Pediatric in vivo [16, 18] In vitro pediatric [33, 37, 38] In vitro adult [32].
Use conventional aerosol device with concurrent HFNC	Not recommended.	Adult in vitro [32] Pediatric in vitro [34]
Nebulizer placement	VMN should be placed at the inlet of humidifier, except when gas flow is extremely low, such as ≤ 0.25 L/kg/min for infants	Pediatric in vitro [25, 30] Adult in vitro [26, 39]
Gas flow setting during trans-nasal aerosol delivery	If possible, titrate HFNC gas flow below the patient’s inspiratory flow	Pediatric in vivo [38, 40] Adult in vivo [42] Pediatric in vitro [23, 33, 38] Adult in vitro [26, 27, 44].
Open mouth breathing during trans-nasal aerosol delivery	When gas flow exceeds patient inspiratory flow, open mouth breathing reduces inhaled dose; when gas flow is below the patient’s inspiratory flow, open mouth breathing could generate higher inhaled dose.	Adult in vitro [26] Pediatric in vitro [37]
Use heliox to deliver aerosol via HFNC	Might be considered for pediatric patients	Adult in vitro study [27] Pediatric in vitro [23]
Use dry gas to deliver aerosol via HFNC	Not recommended	adult in vivo [42]
Using frequent unit doses or infusion pump to deliver continuous albuterol for asthma exacerbation	If possible, use unit dose to deliver albuterol and decrease gas flow during nebulization; return flow to original setting when nebulization is completed. Titrate F_I_O_2_ to maintain SpO_2_ during the periods of flow reduction. If infusion pump has to be used, relative low gas flow and a higher nominal dose could be considered.	Pediatric in vitro [37]
Stable COPD	Standard dose (2.5 mg) of albuterol is sufficient to elicit bronchodilation responses with HFNC gas flow set at 15–20 L/min.	Adult in vivo [9, 10, 12]
COPD exacerbation	Standard dose (2.5 mg) of albuterol as a starting dose with HFNC flow set at 20–30 L/min is recommended during trans-nasal aerosol delivery.	Adult in vivo [9, 10, 12]
Pulmonary hypertension without hypoxemia	HFNC flow set at 5–10 L/min is recommended	Adult in vivo [15]
Pulmonary hypertension with refractory hypoxemia	Titrating HFNC flow at bedside based on patient’s response in order to determine the optimal flow for each individual patient is recommended	Adult in vivo [15]

*HFNC* high-flow nasal cannula, *VMN* vibrating mesh nebulizer, *F*_*I*_*O*_*2*_ fraction of inspired oxygen, *SpO*_*2*_ peripheral capillary oxygen saturation, *COPD* chronic obstructive pulmonary disease

### Asthma exacerbation

During status asthmaticus, HFNC improves work of breathing and reduces carbon dioxide retention [2, 58, 59]. The comfort of aerosol delivery via HFNC makes it an ideal option, particularly for young children [8, 16, 17]. High gas flow setting has some benefits, but it impedes aerosol delivery to the lung. Reduction in HFNC flow from 2.0 to 0.5 L/kg/min resulted in a 21% increase in patient’s work of breathing [60]. However, the inhaled dose by trans-nasal aerosol delivery at the lower gas flow increased by 3- to 12-fold [30, 37, 38, 40]. Thus, reducing the HFNC flow improves aerosol delivery at the slight risk of losing breathing support for short periods, while using unit doses could shorten the duration of treatment. Delivery of 1 mL requires 2–4 min; reducing the gas flow for such a short period should not significantly interfere with work of breathing. We caution that when HFNC flow is reduced, the patient should be closely monitored and F_I_O_2_ increased if needed to maintain a target SpO_2_ [61].

Patients with severe asthma often require larger than conventional bronchodilator doses. Multiple unit doses are delivered more frequently, requiring intensive use of staff resources. Bronchodilators could be delivered using HFNC with an infusion pump and prepared syringe of albuterol. In this scenario, a higher dose could be delivered to the lung by utilizing a relatively low gas flow and a slightly higher nominal dose compared to conventional bronchodilator aerosol delivery techniques [37].

### Stable COPD and COPD exacerbation

HFNC is increasingly utilized for patients with COPD for its effects of washing out dead space and reducing work of breathing [2, 62]. Long-term use of HFNC could reduce the frequency and duration of COPD exacerbations and improve patient’s quality of life [63, 64]. In those studies with stable COPD, HFNC flow was set at 20–25 L/min [63, 64] due to the patient’s low inspiratory flow demand. HFNC gas flow of 15–20 L/min with a standard dose of 2.5 mg albuterol is sufficient to elicit bronchodilator effects [12].

During exacerbation of COPD, higher than usual patient’s inspiratory flow demand requires an increase of flow setting with HFNC. In 12 hypercapnic patients with COPD initially treated with noninvasive ventilation, Rittayamai and colleagues achieved similar work of breathing with HFNC flow set at 30 L/min [65]. Discontinuing HFNC to use a facemask significantly increased patients’ breathing efforts [66]. Therefore, HFNC should not be interrupted to use mask/mouthpiece with JN when acutely ill patients with COPD require aerosol treatments. In this setting, it is appropriate to deliver aerosol via HFNC at 20–30 L/min flow.

### Pulmonary hypertension with/without hypoxemia

In patients with pulmonary hypertension, prolonged continuous inhalation of aerosolized epoprostenol with HFNC is convenient and comfortable for the patient. In the absence of concomitant hypoxemia and with low inspiratory flow demand, low gas flows with HFNC (5–10 L/min) could optimize delivery of inhaled epoprostenol to patients with pulmonary hypertension [15, 27, 29, 42].

When patients with pulmonary hypertension have concomitant hypoxemia, higher gas flow and F_I_O_2_ are required to improve oxygenation by avoiding air entrainment and generating some positive end-expiratory pressure [58, 66]. However, high gas flows reduce trans-nasal delivery of inhaled epoprostenol with a potential loss of efficacy because there is a linear correlation of inhaled dose with improvement in oxygenation [20]. A practical solution would be to titrate HFNC gas flow at the bedside to pulmonary arterial pressure and/or oxygenation [15]. This approach could determine optimal setting for individual patient, based on immediate responses to inhaled epoprostenol.

### Summary

Patients, who did not require HFNC for administering high F_I_O_2_ and who could tolerate reduced gas flow for short periods, could be benefited by decreasing HFNC gas flow to relatively low settings, such as 15–20 L/min for stable adult patients, 20–30 L/min during COPD/asthma exacerbation among adults, and 0.25 L/kg/min for children with asthma. Employing unit doses with high drug concentration could shorten the duration for which flow was reduced. After administration of the unit dose, HFNC gas flow should be returned to its previous setting. For patients receiving HFNC therapy mainly for relief of hypoxemia and who simultaneously require inhaled epoprostenol to improve oxygenation, HFNC gas flow should be carefully titrated at the bedside based on patients’ response to both inhaled epoprostenol and gas flow.

## Future directions

More clinical studies are needed to validate the in vitro findings, such as the impact of gas flow on aerosol delivery via HFNC, and the effective dose at those flow settings [46]. Clinical studies of patient safety are also warranted, particularly the potential for toxicity or harm with off-label use of medication inhaled via HFNC. The use of submicrometer droplets combined with condensational growth technology has shown significant improvements of inhaled dose during trans-nasal aerosol delivery in vitro [67], and its application in clinical practice is awaited.

## Conclusion

Trans-nasal pulmonary aerosol delivery via HFNC is a promising method for continuous administration of medication for prolonged periods, especially for children. However, clinicians must consider the features and limitations of the device, and the patient’s disease severity. There is increasing evidence to support clinical efficacy and safety of trans-nasal pulmonary aerosol delivery via HFNC. Prospective, well-designed studies in appropriate populations of patients are needed to establish the efficacy of this mode of aerosol administration. We provide practical recommendations for employing trans-nasal pulmonary aerosol delivery via HFNC in acutely ill patients.

## Data Availability

Not applicable.

## Abbreviations

HFNC: High-flow nasal cannula
RCT: Randomized controlled trials
ICU: Intensive care unit
COPD: Chronic obstructive pulmonary disease
FEV_1_: Forced expiratory volume in the first second
VMN: Vibrating mesh nebulizer
MDI: Metered dose inhaler
JN: Jet nebulizers
PaO_2_: Partial pressure of arterial oxygen
SpO_2_: Peripheral capillary oxygen saturation
F_I_O_2_: Fraction of inspired oxygen
CI: Confidence interval
